# Relationship between the level of *willingness to learn* about anti-doping and objective knowledge among Japanese university athletes: A cross-sectional study

**DOI:** 10.3389/fspor.2022.955636

**Published:** 2022-08-15

**Authors:** Yuka Murofushi, Yujiro Kawata, Shinji Yamaguchi, Miyuki Nakamura, Yuji Takazawa, Hisashi Naito

**Affiliations:** ^1^Faculty of Health and Sports Science, Juntendo University, Chiba, Japan; ^2^Graduate School of Health and Sports Science, Juntendo University, Chiba, Japan; ^3^Institute of Health and Sports Science & Medicine, Juntendo University, Chiba, Japan

**Keywords:** anti-doping education, anti-doping knowledge, *willingness to learn*, 2021 Code, international standard for education

## Abstract

Previous studies have indicated that athletes' anti-doping knowledge is inadequate. Athletes' willingness to learn about anti-doping (*willingness to learn*) may influence their anti-doping knowledge, but the actual situation is unclear. This study aimed to determine the relationship between athletes' *willingness to learn* about anti-doping and their objective measurement knowledge and explore directions for educational interventions. The eligible participants were 971 male and 802 female university athletes. We used the ALPHA test (12 questions/four choices; passing index: ≥10 points/80% correct answer rate) to assess objective anti-doping knowledge. The *willingness to learn* question was, “Would you like to learn more about anti-doping?” Responses were given on a 4-point scale ranging from 1: strongly disagree to 4: strongly agree. An ANCOVA was conducted with four levels of *willingness to learn* as the independent variable and ALPHA correct answer rate as the dependent variable, adjusting for confounding factors (years of athletic experience and anti-doping education experience). The percentage of athletes (%) and each ALPHA correct answer rate (%) by the level of *willingness to learn* was 1: strongly disagree, *n* = 1.64%, 61.78%; 2: somewhat disagree, *n* = 13.14%, 62.38%; 3: somewhat agree, *n* = 62.94%, 64.08%; 4: strongly agree, *n* = 22.28%, 67.11%. The ALPHA correct answer rates showed significant differences in the main effect by the level of *willingness to learn* [*F*_(3, 1767)_ = 2.873, *p* < 0.05, η^2^ = 0.01], although the effect size was small, and multiple comparisons showed no significant differences between the levels. The results indicated that the ALPHA correct answer rate did not reach 80% even for the “strongly agree” level of *willingness to learn*, suggesting that information on anti-doping may be inadequate. The need to provide sufficient educational content to improve knowledge was evident.

## Introduction

Doping is considerably more prevalent than is commonly imagined, and the effects extend far beyond the scale of the Olympics doping incidents to date (Yesalis and Bahrke, 2002). As a background to establishing a doping culture, it has been pointed out that one of the factors behind rampant doping is the involvement of experienced professional athletes in teaching young amateur-level athletes how to dope (Lentillon-Kaestner and Carstairs, 2010). In other words, doping behavior begins at a young age and amateur level, which is a critical period for accepting or rejecting doping. Understanding the factors contributing to an athlete's decision to dope will encourage the deliberation of preventive anti-doping strategies. Kegelaers et al. (2018) found that an athlete's practical decision of whether to use a prohibited substance or method is extraordinarily complex and dynamic. Many multi-level triggers and deterrents influence, including athletic, psychological, social, financial, and policy. Also, social, financial, or policy is influenced by a wide range of multi-level incentives and deterrents, as well as career transitions. In light of these factors, it is necessary to focus on athletes' education in the stages prior to athletic success to prevent doping. In this context, educators must improve athletes' anti-doping knowledge to prevent intentional/unintentional doping, enabling athletes to behave ethically from a young age. Improving athletes' understanding of accurate anti-doping information and their *willingness to learn* are essential to prevention efforts.

In sports history, anti-doping rule violations (ADRVs) have been detected among elite athletes (Todd and Todd, 2001). ADRVs persist, although doping control has been implemented since the 1960s to deter doping behaviors (Todd and Todd, 2001), such as athletes' use of prohibited substances and methods (Müller, 2010; Overbye et al., 2015). The number of ADRVs reported internationally varies significantly from country to country. Some countries have <10 cases yearly, while others have more than 100 cases (World Anti-Doping Agency, 2021a). So far, intelligence aspects, such as the expansion of the scale of doping control and the ability to analyze samples, have been improved to deter ADRVs. Unfortunately, this has not led to a significant reduction in ADRVs (Laure and Binsinger, 2007; Martínez-Sanz et al., 2017). A rough estimate of doping prevalence in elite sports has been provided through previous studies (Pitsch and Emrich, 2012). However, concerns have been raised that doping control is insufficient to estimate true doping prevalence, and the deterrent approach of doping testing alone is reaching its limits (de Hon et al., 2015). Considering this situation, some studies that have focused on countries (or sports) with a high number of ADRVs in the past have been negative toward anti-doping education (ADE), and some have opined that improving anti-doping knowledge and providing information for prevention do not necessarily prevent doping (Peretti-Watel et al., 2005; Lentillon-Kaestner et al., 2012; Morente-Sánchez and Zabala, 2013).

In contrast, an education provision was included in Article 18 of the first Code (World Anti-Doping Agency, 2021b), an internationally well-known fact recognizing ADE's importance. Furthermore, in 2014, the World Anti-Doping Agency (WADA) declared that ADE was at the center of its anti-doping strategic plan (World Anti-Doping Agency, 2016), while the roadmap at the time of the 2015 Code establishment (Dvorak et al., 2014) focused more on ADE and awareness of preventing doping.

Nevertheless, in the period leading up to the consultation for developing the 2021 Code (World Anti-Doping Agency, 2019), there was insufficient discussion to establish standards to promote ADE internationally. Moreover, previous ADE programs to increase athletes' anti-doping knowledge were relatively ineffective, and the program's design vulnerabilities were also noted (Woolf, 2020). However, the deterrent measures to discourage doping by considering the severity and threat of sanctions (Engelberg et al., 2014; Ntoumanis et al., 2014) when an ADRV results from a doping test have not adequately reduced the occurrence of ADRVs in real situations. These trends and backgrounds have further reinforced the importance of adopting a preventive perspective and an educational approach to doping. This perspective has led to an international education policy that ADE should begin at as young an age as possible based on the values of sport.

Furthermore, “an athlete's first experience with anti-doping should be through education rather than doping control” (Dvorak et al., 2014; World Anti-Doping Agency, 2021c). In this vein, the International Standard for Education (ISE) was established for the first time when the 2021 Code came into effect. The ISE mandates education for the deterrence and precaution of doping (World Anti-Doping Agency, 2021c). The ISE is an international standard that considers the diversity of educational approaches and differences in cultural backgrounds and requires the cooperation of different National Anti-doping Organizations (NADOs) worldwide.

The purpose of the ISE is to conduct ADE with awareness of both intentional and unintentional doping precautions. In establishing the ISE, the importance of providing education and awareness based on scientific evidence to help promote the precaution of doping, a multifaceted and complex phenomenon, was further recognized (Sipavičiute et al., 2020). “Awareness-raising” is one of the four elements of ISE education. An approach that enables athletes to recognize and act independently is indispensable. A previous study showing an attempt to intervene with an ethical decision-making training program for young elite athletes reported a significant decrease in attitudes toward doping after the program (Elbe and Brand, 2016). Furthermore, Hurst et al. (2020) evaluated the effectiveness of a multi-step ADE program covering the Code. The results showed that the unintentional doping measure remained positive up to 3 months after the program. Furthermore, athletes' anti-doping knowledge increased, and their subsequent use of sports supplements and beliefs in their effectiveness decreased. In support of these findings, particular perspectives in cognitive research suggest that psychological processes related to doping are particularly associated with attitudes and attitude change (Hauw and McNamee, 2015). Therefore, athletes' attitudes toward doping are essential psychological predictors of doping intentions. However, ongoing ADE is necessary to reduce the risk of unintentional doping, as its ineffectiveness in preventing intentional doping in the long term suggests the need for a long-term ADE approach (Hurst et al., 2020).

Athletes who experienced ADE, which comprehensively focused on personal development and competency, are more supportive of anti-doping policies than those who receive basic ADE (Barkoukis et al., 2022). Furthermore, athletes who have experience with comprehensive ADE have reported significantly higher perceived legitimacy, trustworthiness, and obedience intent. In other words, ADE and its environment can influence athletes' willingness to support anti-doping policies. This finding suggests that ADE programs must focus on the integrity of the sport and the individual athlete. An extensive survey assessing perceptions of ADE among elite youth athletes found that those with selective educational experiences had higher perceptions of the usefulness and reliability of ADE than athletes who were provided with information-based ADE (Gatterer et al., 2021). Comprehensive ADE is consistent with claims strongly associated with trustworthiness and legitimacy (Blank et al., 2022). Most athletes desire clean sporting behavior free of doping and need ADE to reinforce the commitment to clean sport rather than to prevent doping (Blank et al., 2022), so a multifaceted ADE goes beyond information-based knowledge to promote compliance with the Code and to develop decision-making and practical skills.

Previous studies have indicated that anti-doping knowledge among athletes when measured objectively, is inadequate (Fürhapter et al., 2013; Muwonge et al., 2015; Kim and Kim, 2017; Murofushi et al., 2018). In particular, athletes have a limited understanding of medical knowledge, such as doping's side effects and roles and responsibilities (World Anti-Doping Agency, 2021c). Surveys of ADE among Japanese university athletes have revealed that doping tests alone are insufficient to acquire appropriate knowledge. A lack of knowledge persists in all individual competition levels, including *recreational, national-level*, and *international-level athletes* (Murofushi et al., 2018). However, the same study found that athletes with more ADE experience had significantly higher knowledge than those with less ADE experience, demonstrating the usefulness of ADE implementation. A survey of ADE preferences across multiple countries revealed that most athletes were less willing to undergo ADE, with women and older athletes or athletes from certain countries more willing to learn about anti-doping (Skoufa et al., 2022). Previous studies have shown that willingness or motivation to learn is essential in enhancing academic performance in studies and other activities (Schiefele, 1991). These findings suggest that athletes' *willingness to learn* may have at least some influence on their anti-doping knowledge. However, no studies to date have examined the relationship between *willingness to learn* and anti-doping knowledge. In promoting ISE-based ADE, it is crucial to understand the actual status of athletes' predisposition to accept ADE and to capture the influence of *willingness to learn* on anti-doping knowledge.

Based on the above, this study aimed to determine the relationship between Japanese university athletes' *willingness to learn* about anti-doping and their objective anti-doping knowledge. This study hypothesized that a higher *willingness to learn* about anti-doping would be associated with higher anti-doping knowledge. Most previous anti-doping studies have focused on top athletes capable of dealing with doping control. Understanding the actual status of anti-doping *willingness to learn* at all individual competition levels is expected to provide new perspectives for future ADE interventions and valuable information for promoting international education based on ISEs.

## Materials and methods

### Research design

This study used a cross-sectional design.

### Participants

The eligible participants were 1,773 Japanese university athletes (20.4 ± 1.3 years old) affiliated with Japanese sports universities, involving 971 men (19.6 ± 1.1 years old, 54.80%) and 802 women (20.6 ± 1.2 years old, 45.20%). The survey was limited to sports disciplines in which doping control is implemented by signing the WADA Code. All individual competition levels were included in the survey.

#### Participants' characteristics

We collected the following demographic information from participants, including sex, age, athletic event (open skill and closed skill), individual competition level [recreational athletes (district and prefecture level), national-level athletes, and international-level athletes], years of competition experience (≤ 5, 6–10, ≥ 11 years), doping control experience, and anti-doping education experience. At the time of the survey, there was no consistent anti-doping education in Japan. Therefore, this study asked respondents to self-report whether they had received anti-doping education.

### Measures

#### Anti-doping willingness to learn scale

The question on anti-doping *willingness to learn* was, “Would you like to learn more about anti-doping?” The questionnaire surveyed responses on the level of *willingness to learn* on a 4-point Likert scale ranging from 1: strongly disagree to 4: strongly agree. Higher scores indicated a greater *willingness to learn*. Each of the four responses was positioned as a level of *willingness to learn*.

#### Measurement of anti-doping knowledge (ALPHA test)

This study measured university athletes' anti-doping knowledge using the ALPHA test, a questionnaire that objectively measures anti-doping knowledge (Murofushi et al., 2018). The ALPHA test is a multiple-choice test consisting of 12 questions with four response options. One response is selected for each question. For each of the 12 correct answers, the final evaluation score was the sum of the numerical values, which were 1 for correct answers and 0 for incorrect answers (score range: 0–12). The ALPHA pass index of 10 points (80% or higher) was used as the evaluation index. The pass index for each ALPHA question was defined as 80%, which is the total score index of the ALPHA test.

### Procedure

The research was carried out after obtaining approval from the research society ethics committee of the Faculty of Health and Sports Science and the Graduate School of Health and Sports Science, Juntendo University, Japan (Ethics Approval Number: 2020-15). The study was conducted from October 2017 to January 2020, before the ISE was established. Seven Japanese universities with sports departments were contacted by e-mail and explained the purpose of our study. After receiving permission from each university to conduct the survey, each university was visited at the time and date confirmed by them and surveyed the participants during that period. The times specified for survey implementation were before and after classes were in session, so no particular impact on the survey participation rate occurred. In face-to-face meetings, we explained the purpose of the study and that participation in the survey was voluntary. Participants were informed that because of the anonymous nature of the survey, data could not be excluded after collection. They were also informed how to complete the survey and that they could withdraw their consent to the study at any time and discontinue participation without prejudice. After the participants read the study description and gave informed consent, they answered the questionnaire, and the survey form was collected by the researchers immediately after being completed. Data from 2,034 participants was collected. From these responses, 261 participants (12.8%) were excluded due to missing or incomplete data, for example, incomplete data. The final sample included 1,773 participants.

### Analytical design and statistical processing

#### Participants' characteristics

Descriptive statistics were used to calculate the percentage of sample size for each of the participant characteristics.

#### *Willingness to learn* and ALPHA scores by participants' characteristics

The *willingness to learn*, ALPHA scores, and standard deviations were calculated using a comparative analysis of means for participant characteristics. We also calculated the correct answer rates regarding the ALPHA test.

#### Comparison of ALPHA scores by level of *willingness to learn*

##### Identification of confounding factors affecting *willingness to learn* and ALPHA scores

The confounding factors that influenced the *willingness to learn* scale were identified and ALPHA test scores. Previous studies (Murofushi et al., 2018) found that athlete category and anti-doping education experience were associated with ALPHA scores but not sex, years of competition experience, or doping control experience. In this study, we examined whether these were confounding factors. A MANOVA was conducted using sex, individual competition level, years of competition experience, doping control experience, and anti-doping education experience as independent variables, and the *willingness to learn* and ALPHA scores as dependent variables.

##### Comparison of *willingness to learn* levels and ALPHA scores

First, the mean and standard deviation of ALPHA scores by level of *willingness to learn* were calculated. Next, the primary analysis was performed on the relationship between *willingness to learn* level and ALPHA scores. Finally, an ANCOVA was conducted by setting the level of *willingness to learn* as the independent variable, ALPHA score as the dependent variable, and confounding factors as the covariates. For subsequent multiple comparisons, the Bonferroni correction was applied.

#### Comparison of correct answer rate by the level of *willingness to learn* for each ALPHA question

For each of the 12 ALPHA questions, the percentage of correct answers by the level of *willingness to learn* was calculated using the comparative analysis of means. For each of the 12 ALPHA question's correct answer rates, the percentage of correct answers by the level of *willingness to learn* was calculated using the comparative analysis of means. Next, to identify confounding factors affecting ALPHA scores, a MANOVA was conducted. Sex, individual competition level, years of competition experience, doping control experience, and anti-doping education experience are independent variables, and each ALPHA question's correct answer rates are dependent variables. An ANCOVA was conducted by setting the level of *willingness to learn* as the independent variable, ALPHA score as the dependent variable, and confounding factors as the covariates. For subsequent multiple comparisons, the Bonferroni correction was applied.

### Statistical processing

The effect size of each analysis was calculated, and the magnitude of the effect size was determined based on the criteria suggested by (Cohen, 1988), where small = 0.01, medium = 0.06, and large = 0.14. The significance level for all analyses was set to <5%. Statistical analysis was performed using IBM SPSS Statistics 28.0.

## Results

### Participants' characteristics data

Table 1 shows participants' characteristics. The proportions of athletes by individual competition levels were as follows: 55.60% (*n* = 987) recreational athletes (district and prefecture-level), 41.50% (*n* = 735) national-level athletes, and 2.90% (*n* = 51) international-level athletes. Athletes with open skill events accounted for 29.72% (*n* = 526) of the sample, and their 24 events included athletics, swimming, and gymnastics. The closed skill event group was 67.96% (*n* = 1,205), and their 23 events included football, baseball, and basketball. The percentage of athletes with experience in doping control was 2.40% (*n* = 42). Athletes with anti-doping education experience accounted for 53.30% (*n* = 945) of the participants, more than half of the total.

**Table 1:** Participants' characteristics.

**Category**	**n**	**%**
**Sex**		
Men	971	54.80
Women	802	45.20
**Individual competition level**	***n***	**%**
Recreational athletes (district)	488	27.50
Recreational athletes (prefecture)	499	28.10
National-level athletes	735	41.50
International-level athletes	51	2.90
**Competition duration (years)**	***n***	**%**
1–5	570	32.12%
6–10	589	33.22%
≥11	614	34.63%
**Doping control experience**	***n***	**%**
Experienced	42	2.40
Non-experienced	1731	97.60
**Education experience**	***n***	**%**
Experienced	945	53.30
Non-experienced	828	46.70
**Athletic event (Total 47 sports)**	***n***	**%**
Closed skill event	526	29.67%
Open skill event	1205	67.96
Non-response	42	2.37

*Closed skill event: Athletics, swimming, gymnastics and others. Open skill event: football, baseball, basketball and others*.

### Calculation of *willingness to learn* and ALPHA scores/correct answer rates by participant characteristics

The *willingness to learn* scores (SD) and ALPHA scores (SD)/correct answer rates by participant characteristics are shown in Table 2. The mean overall *willingness to learn* score was 3.06 ± 0.65 points (~3), or “somewhat agree.” Approximately 85% of the participants had a *willingness to learn* score of “somewhat agree” or higher. The mean overall ALPHA score and correct answer rate were 7.74 ± 2.40 points (63.3%), <80% as a passing index. In the distribution of ALPHA scores, a quarter (*n* = 443, 24.99%) of the participants scored more than 80% as passing index.

**Table 2:** Comparison of anti-doping *willingness-to-learn* score and ALPHA score by participants' characteristics.

**Category**	**Classification**	***n***	**(*n* %)**	***Willingness to learn*** **score**					**ALPHA score**					
				**Mean**	**SD**	***p***	**η^2^**	**MCT**	**Mean**	**SD**	**Correct answer rate (%)**	***p***	**η^2^**	**MCT**
Sex	Men	971	54.77	3.06	0.63	0.967	<0.01	—	7.60	2.55	63.3	0.785	<0.01	—
	Women	802	45.23	3.06	0.67				7.91	2.17	66.0			
Years of competition experience (years)	≤ 5	570	32.15	3.13	0.63	0.014	0.01	^**^≥11 < ≤ 5	8.07	2.36	67.3	0.515	<0.01	—
	6–10	589	33.22	3.06	0.65				7.57	2.36	63.1			
	≤ 11~	614	34.63	3.00	0.65				7.59	2.40	63.3			
Individual competition level	Recreational athletes (District)	488	27.52	3.03	0.64	0.316	<0.01	—	7.98	2.38	66.5	0.325	<0.01	—
	Recreational athletes (Prefectural)	499	28.14	3.13	0.64				7.80	2.38	65.0			
	National-level athletes	735	41.46	3.02	0.64				7.48	2.40	62.3			
	International-level athletes	51	2.88	3.18	0.68				8.59	1.95	71.6			
Doping control	Experienced	42	2.37	3.21	0.65	0.337	<0.01	—	8.00	2.12	66.7	0.324	<0.01	—
	Non-experienced	1,731	97.63	3.05	0.65				7.73	2.39	64.4			
Anti-doping education Experience	Experienced	945	53.30	3.07	0.64	0.179	<0.01	—	8.11	2.19	67.6	0.011	<0.01	***Non-experienced < Experienced
	Non-experienced	828	46.70	3.05	0.65				7.32	2.53	61.0			

**p <0.01, ***p <0.001. MCT, multiple comparison testing. Willingness to learn score obtained using the anti-doping willingness to learn question, “Would you like to learn more about anti-doping?” and selected option based on a 4-point Likert scale ranging from 1: strongly disagree to 4: strongly agree (score range: 1–4). ALPHA score range: 0–12 points. ALPHA passing index ≥ 80%. ALPHA Correct answer rate (%): ALPHA correct answer rate by demographic data.

### Comparison of ALPHA scores by level of *willingness to* learn

#### Identification of confounding factors affecting *willingness to learn* and ALPHA scores

We identified the confounding factors affecting *willingness to learn* and ALPHA scores. A MANOVA was conducted with the independent variables set as sex, individual competition level, years of competition experience, doping control experience, and anti-doping education experience, and the dependent variables set as the *willingness to learn* and ALPHA scores. Consequently, extraneous variables were identified between the years of competition experience and *willingness to learn* scores and between anti-doping education experience and ALPHA scores (*p* < 0.05; Table 2). Therefore, these variables were considered confounding factors in the subsequent analysis.

#### Comparison of *willingness to learn* levels and ALPHA scores

**Figure 1:**
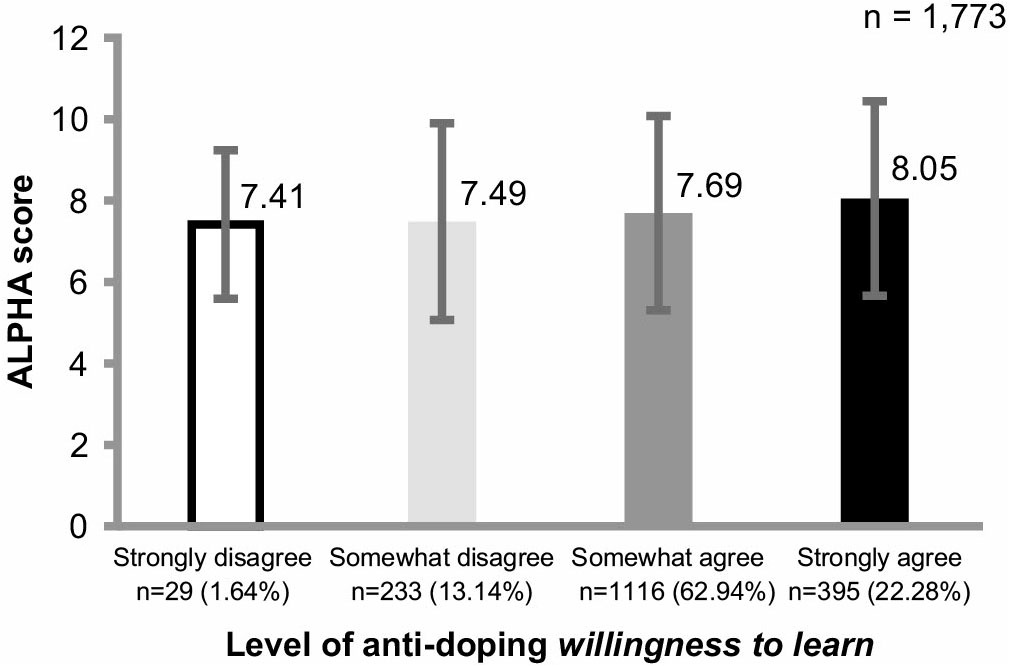
Comparison of anti-doping *willingness to learn* level and ALPHA scores. *n* (%) is the percentage of the total number of athletes. The *Willingness to learn* score was obtained using the anti-doping *willingness to learn* question, “Would you like to learn more about anti-doping?” and the selected option based on a 4-point Likert scale ranging from 1: strongly disagree to 4: strongly agree (score range: 1–4). ALPHA score: Score range 0–12 points. ALPHA passing index is ≥10 points/correct answer rate ≥80%. The ALPHA correct answer rates showed significant differences in the main effect by the level of *willingness to learn* (*p* < 0.05, η^2^ = 0.01), although the effect size was small and multiple comparisons showed no significant differences between the levels.

The ALPHA mean scores by *willingness to learn* levels were strongly disagree: 7.41 ± 1.82 points, *n* = 29; somewhat disagree: 7.49 ± 2.42 points, n = 23; somewhat agree: 7.69 ± 2.39 points, *n* = 1,116; and strongly agree: 8.05 ± 2.39 points, *n* = 395. An ANCOVA to compare the ALPHA correct response rates by *willingness to learn* levels was performed. The independent variable included the *willingness to learn* levels, the dependent variable was ALPHA correct answer rate, and the covariates were years of competition experience and anti-doping education experience. The analysis showed no significant interaction between ALPHA correct answer rates by *willingness to learn* levels (*F* [4, 1764] = 0.537, *p* < 0.709, η^2^ <0.01; see Figure 1). There was a significant difference in the main effect (*F* [3, 1767] = 2.873, *p* < 0.05, η^2^ = 0.01). There were no significant ALPHA score differences in the pairwise comparison between *willingness to learn* levels.

### Comparison of the correct answers by the level of *willingness to learn* for each ALPHA question

Table 3 compares correct answer rates by the level of *willingness to learn* for each ALPHA question. Significant differences were found in Q2 “What is the purpose of the World Anti-Doping Code?” [*F*_(3, 1769)_ = 2.985, *p* < 0.030, η^2^ = 0.01] (with no confounding factors) and Q12 “What is the requirement for laboratories that analyze blood or urine samples for doping control?” [*F*_(3, 1768)_ =3.175, *p* < 0.023, η^2^ = 0.01] (confounding factors for anti-doping education experience; *p* < 0.05). For Q2, the correct answer rate for strongly agree was significantly higher than that for somewhat agree (*p* < 0.05). For Q12, the correct answer rate for strongly agree was significantly higher than those who answered strongly disagree (*p* < 0.05).

**Table 3:** Correct answers rate by the level of *willingness-to-learn* for each ALPHA question.

**No**.	**ALPHA question content (Correct answer rate %)**	**Anti-doping** ***willingness-to-learn*** **level**					***p***	**η^2^**	**MCT**
		**All** ***N* = 1,773**	**Strongly disagree** ***n* = 29**	**Somewhat disagree** ***n* = 233**	**Somewhat agree** ***n* = 1,116**	**Strongly agree** ***n* = 395**			
1	What is the philosophy behind anti-doping?	85.6	93.1	85.0	84.8	87.6	0.349	0.01	—
2	What is the purpose of the World Anti-Doping Code?	50.5	48.3	46.8	49.0	57.0	0.030	0.01	*Somewhat agree < strongly agree
3	What is the Prohibited List?	76.1	79.3	72.5	75.9	78.5	0.417	<0.01	—
4	What are the side effects of using anabolic steroids?	36.1	41.4	33.0	36.1	37.5	0.657	<0.01	—
5	What does TUE stand for?	64.1	48.3	60.5	64.2	67.1	0.112	<0.01	—
6	How can an athlete with a medical condition decide whether to take a medication?	72.5	65.5	76.0	71.5	73.7	0.390	<0.01	—
7	Who is responsible for the substances found in an athlete's body?	55.8	58.6	52.8	55.8	57.5	0.849	<0.01	—
8	What condition allows an athlete to refuse to be tested?	87.1	86.2	86.7	86.7	88.6	0.806	<0.01	—
9	When must an athlete be notified of an upcoming test?	55.4	37.9	54.1	54.7	59.5	0.083	<0.01	—
10	When do athletes have to tell their National Anti-Doping Organization where they will be living, training and competing?	43.7	34.5	45.1	43.4	44.3	0.733	<0.01	—
11	What are the athlete's right when a positive test is returned?	49.1	51.7	45.5	48.8	51.9	0.465	<0.01	—
12	What is the requirement for laboratories that analyze blood or urine samples for doping control?	64.2	62.1	55.8	64.6	68.1	0.023	0.01	*Strongly disagree < Strongly agree

*p <0.05. MCT, multiple comparison testing. ALPHA score: Score rage 0–12 points. ALPHA passing index is ≥10 points/correct answer rate ≥80%. Willingness to learn score obtained using the anti-doping willingness to learn question, “Would you like to learn more about anti-doping?” and selected option based on a 4-point Likert scale ranging from 1: strongly disagree to 4: strongly agree (score range: 1–4). All: correct answer rate for the total number of participants. 1. Strongly disagree, 2. Somewhat disagree, 3. Somewhat agree, 4. Strongly agree: correct answer rate by level of willingness to learn. The level of willingness to learn with the highest correct answer rate for each item is shown in bold and underlined.

The question with the highest number of correct answers was Q8 “What condition allows an athlete to refuse to be tested?” followed by Q1 “What is the philosophy behind anti-doping?” having a correct answer rate of ~85% at all *willingness to learn* levels. The lowest correct answer rate was for Q4 “What are the side effects of using anabolic steroids?” at the “somewhat disagree” level. The next question with the lowest correct answer rate was, Q10 “When do athletes have to tell their National Anti-Doping Organization where they will be living, training and competing?” The “strongly disagree” level had the lowest correct answer rates of all the *willingness to learn* levels. The “strongly agree” level had the highest correct answer rates to seven of the 12 items (Questions 2, 5, 6, 8, 9, 11, and 12) of all the *willingness to learn* levels.

## Discussion

### *Willingness to learn* and alpha scores by participant characteristics

The present study revealed a previously unexplored aspect of *willingness to learn* about anti-doping among Japanese university athletes. The participants' *willingness to learn* had a mean score of “*somewhat agree*” in all categories and by the participant characteristics. Approximately 85% of the participants were at the “*somewhat agree*” level or higher. Regarding *willingness to learn*, a survey of soccer players in three countries reported that many athletes were not optimistic about ADE (Skoufa et al., 2022). The percentage of athletes who were *willing to learn* was more than 60% by nationality and other attributes, and by age, the percentage was lower among younger athletes (in the 20% range) while women and older athletes who had been competing for a long time showed higher *willingness to learn*.

These findings suggest that the nature of the educational programs may influence future ones provided through NADOs and National Federations (NFs). Athletes may perceive ADE as a formality of the learning process rather than a crucial means of learning about doping (Johnson et al., 2013). There is no denying that athletes experience some aspects of ADE efforts as a formality. In light of the anti-doping framework proposed by Donovan et al., a significant factor in athletes' attitudes and behaviors regarding doping relates to how athletes perceive the legitimacy of governing bodies and enforcement agencies related to anti-doping (Donovan et al., 2002).

Based on these studies, it is possible that the type of formal ADE that athletes receive may affect their *willingness to learn*. Although all individual competition levels surveyed participated in this study, it is worth noting that more than half already had experience with ADE, and 85% indicated a *willingness to learn*. Although ADRVs tend to be relatively rare in Japan, factors including respect, *willingness to learn* about anti-doping, and having ADE experience may also be related to the level of awareness. Further research is needed on the background search to support these findings. The mean ALPHA score in a previous study was 7.75 points (64.5% correct) (Murofushi et al., 2018), but the result of the present study was 7.74 points (63.3% correct), both of which are lower than the pass index of 10 points. In addition, some studies (Murofushi et al., 2018) found that the national level had the lowest mean score (7.48 points) and the international level had <10 points (8.59 points). Several other studies have also indicated that athletes' anti-doping knowledge is low (Fürhapter et al., 2013; Muwonge et al., 2015; Kim and Kim, 2017).

Prior to establishing the ISE, ADE targeted elite athletes (Kim et al., 2011; Morente-Sánchez and Zabala, 2013; Muwonge et al., 2015; Murofushi et al., 2018) at the highest levels of their respective sports at the national or international level (Dunn et al., 2010). However, there remained a concern that targeting elite athletes and educating them to prepare for doping tests alone would not deter doping. The adequacy of current ADE practices needs to be examined, as anti-doping knowledge among *national*/*international-level athletes*, who are expected to have more ADE opportunities than recreational athletes, is still inadequate. These factors need to be verified further, but national/international-level athletes with relatively more ADE experience may perceive themselves as having enough knowledge. Therefore, it is necessary to understand whether there is a discrepancy between subjective and objective knowledge. Further consideration of educational approaches that match subjective and objective knowledge levels is needed to prevent ADRVs. Such an approach is expected to reduce the likelihood of ADRV.

### Relationship between the level of *willingness to learn* about anti-doping and ALPHA score

This study revealed the relationship between the *willingness to learn* about anti-doping and objective measurement knowledge among Japanese university athletes. Comparing the relationship between *willingness to learn* and ALPHA scores, there was a significant main effect of ALPHA scores between the levels of *willingness to learn*. The effect size, indicating the strength of the relationship between the variables, was η^2^ = 0.01, as the small level (η^2^ = 0.01). However, multiple comparison analyses showed no significant differences in ALPHA scores between levels of *willingness to learn*. Hence, the results did not indicate that athletes with a higher level of *willingness to learn* were more knowledgeable about anti-doping. Furthermore, it cannot be overlooked that the average ALPHA score was <10 points on the acceptance index at all levels of *willingness to learn*. From the perspective that motivation and *willingness to learn* are some of the crucial factors that enhance academic achievement (Schiefele, 1991) and that a higher *willingness to learn* about anti-doping predicts increased knowledge, our findings reject the hypothesis of this study.

There are two possible explanations for these results. The first point is the lack of information about anti-doping and the teaching content. In the present study, 85% of the athletes' *willingness to learn* about anti-doping involved “somewhat agree” or more as the response. Although surveys in other countries have reported less favorable attitudes toward ADE (Skoufa et al., 2022), a certain number of highly motivated athletes were present in this study for comparison. Nevertheless, there was no difference in general knowledge between the groups that answered “somewhat disagree” or less and those that answered “somewhat agree” or more. The present analysis adjusted the effect of ADE frequency. However, athletes may already have received ADEs with deficient learning content, reducing the importance of educational frequency. Hence, even if the athletes had a strong *willingness to learn*, inadequate ADE may have been the failure factor to reach the ALPHA score passing index. Therefore, for athletes who have some *willingness to learn*, ADE that covers the information and content necessary to enhance their knowledge may currently be lacking.

The discrepancy between willingness and knowledge is also evident in a prior survey of elite athletes asking about their ADE experience (Somerville and Lewis, 2005). Regarding the provision of anti-doping information, athletes perceive that they receive the information they need to avoid ADRVs. There is also an ambitious belief that athletes need more frequent opportunities to be reminded of anti-doping rules. The anti-doping authorities overseeing ADE should therefore increase ADE opportunities. Meanwhile, athletes show divergence in knowledge about anti-doping (Somerville and Lewis, 2005); multiple studies have shown that athletes do not have sufficient knowledge (Somerville and Lewis, 2005; Fürhapter et al., 2013; Muwonge et al., 2015; Kim and Kim, 2017; Murofushi et al., 2018). When there is a discrepancy in the amount of objective knowledge measured against the *willingness to learn*, erroneous or biased knowledge may factor into the present study results. This erroneous knowledge could be related to doping risk. Providing appropriate information so that the Code can be correctly identified and applied to actual behavioral decisions could improve the risk perception of doping.

The second point relates to the athletes' ADE experience. In the past, ADE has been defined as an educational pool by International Federations and Code signatories, such as Registered Testing Pool (RTP)/Testing Pool (TP) and other international-level competitors who submit their whereabouts information and focus on athletes. Education of others has been voluntary, and ADE has been designed for athletes most likely to be tested (Kim et al., 2011; Ozkan et al., 2020; Gatterer et al., 2021; Barkoukis et al., 2022; Skoufa et al., 2022). Many ADE programs have focused on health education and other information such as prohibited substances, prohibited methods, and other topics to deal with doping control; Japan's ADE is no different. Although ADE is expected to change athletes' attitudes toward doping and deter their intent, it has had minimal effect on actual persistence or behavioral aspects.

On the flip side, although comprehensive ADE programs are more supportive of anti-doping policies and are expected to reduce the risk of unintentional doping (Barkoukis et al., 2022), the sustainability of ADE effects has its challenges (Hurst et al., 2020). Meanwhile, evidence suggests that including psychological and other aspects of anti-doping interventions can reduce doping in athletes (Kavussanu et al., 2021). Moreover, an approach that examines the validity of athletes' belief systems and encourages them to think critically about the use of prohibited substances in sports would be practical (Sipavičiute et al., 2020). The learning content of education for non-athletes, as stipulated in the education pool, is left to the provider. It is impossible to ascertain the actual educational content learned by the athletes accurately. Therefore, it is unclear whether the athletes in this study were able to pick up sufficient information in their education to date. In future studies, researchers should conduct surveys that understand the specific ADE topics experienced by athletes and their *willingness to learn*.

Incidentally, doping control opportunities for university athletes in Japan are mainly present at nationally competitive levels and above, such as national championships and intercollegiate games organized by each sports organization. Furthermore, among university students, most athletes who compete in international competitions (e.g., area competitions, Asian Games, Olympics, and World Games) are likely to have doping control experience. However, only about 2.5% of Japanese university athletes experience testing (Murofushi et al., 2018). Furthermore, according to the Japan Anti-Doping Agency's discipline panel report, the total number of ADRVs in Japan from 2007 to 2022 was 87 (a mean of six per year), which means that ADRVs in Japan are extremely rare according to international comparison (World Anti-Doping Agency, 2021a). Given this background, many athletes in this study may not have fully grasped the link between themselves and anti-doping. If they are less likely to be subject to doping tests and do not see themselves as associated with doping, they may develop a sense of otherness regarding anti-doping. Therefore, it is necessary to consider multiple ADE interventions that address these backgrounds. The ISE, developed for the first time in the 2021 Code, articulates the policy that an athlete's first experience with anti-doping should be education before doping control (World Anti-Doping Agency, 2021c). Despite the costs involved, efforts to deter athletes from doping by detecting prohibited substances through doping tests are not fully effective in preventing doping. Most doping-related studies of young athletes often focus on athletes engaged in authentic athletic activities. Laure and Binsinger (2007) focused on young athletes, including those at the pre-adolescent recreational level, and measured the prevalence of doping through a 4-year cohort study. The study found that not only did the rate and frequency of the use of banned substances increase significantly with increasing grade level, but users also had significantly higher levels of trait anxiety than unused athletes. These facts should begin to shape athletes' tolerance and perception of banned substances at least before puberty, so it is necessary to begin providing ADEs from this age. Therefore, there is a need to shape attitudes toward doping in younger generations (Codella et al., 2019). It is imperative to recognize that doping testing alone is insufficient to prevent doping and that education is the only way to minimize doping culture and reduce cases in the mid-to-long term (Morente-Sánchez and Zabala, 2013). In WADA's Athlete Vulnerabilities Research Report, athletes and support staff recognized the need for educational programs covering many ADE topics (World Anti-Doping Agency, 2022). Promoting education to improve practical anti-doping knowledge will correctly identify the rules outlined in the Code and ensure that athletes act accordingly. Future research should further validate the development of preventive anti-doping education programs and educational interventions, considering the pitfalls of intentional and unintentional doping.

### Correct answer rate by the level of *willingness to learn* for each ALPHA

The questions regarding the purpose of the Code and the analytical laboratories for doping test specimens both had significantly higher correct answer rates with a willingness to learn at the level of strongly agree. These results inferred that the analytical laboratories for doping tests have a high level of understanding of the direction of the rules aimed by the Code, including their right as athletes to be provided with a legitimate testing process. The ALPHA correct answer rate by *willingness to learn* was approximately 85% or higher for two items: the basic concept of anti-doping (Q1) and the responsibility to undergo doping testing (Q8), at all levels of *willingness to learn*. In contrast, the side effects of anabolic steroids (Q4) and information on whereabouts (Q10) tended to have the lowest correct answers. These results were comparable to previous studies (Murofushi et al., 2018); in particular, knowledge of questions regarding side effects remained low among university athletes, regardless of their *willingness to learn*. Concerning whereabouts information, the questions were unlikely to be directly addressed by ~90% of the university athletes in this study. The rule is that RTP/TPs are expected to address international-level athletes (World Anti-Doping Agency, 2021b). This standard is thought to be the reason for the lack of ADE opportunities linked to the correct answer rates. Therefore, it is necessary to examine the actual knowledge situation in detail and take necessary ADE measures during future ADE limited to educational pools that submit a place of residence.

Concerning the side effects of doping, while not applicable to all prohibited substances and methods, the possibility of short- or long-term effects can be inferred. Although the drugs used by athletes to dope are considered high doses (Reardon and Creado, 2014), the proper verification is not realistic due to the ethical issues involved in replicating research studies. Predictions are supposed to be made from empirical observations, user reports, and effects in patients prescribed the substance in clinical practice. Morente-Sánchez and Zabala (2013) reviewed athletes' knowledge and awareness of adverse effects of performance-enhancing substances from multiple surveys, the most common being just over 50%. However, surveys that measure knowledge objectively, as this survey does, are scarce, and the current climate is littered with studies that ask about subjective knowledge and perceptions. Therefore, there may be a discrepancy compared to actual knowledge, and it should be assumed that knowledge may be lower than expected. Meanwhile, there are reports that younger athletes are more focused on short-term performance-enhancing effects and do not pay attention to the health effects of long-term use of banned substances (Lentillon-Kaestner et al., 2012). Even if they understand, the dilemma remains that knowledge does not deter doping.

The educational topics the participants in this study have studied remain unclear. In Japan, the reality is that ADE has focused on content to prevent the unintentional use of prohibited substances and methods. However, limited information has been provided regarding the side effects of doping (Murofushi et al., 2018). Hence, objective knowledge may remain low due to the lack of information on adverse effects. There are few violations involving the intentional use of banned substances or methods reported in the sports field in Japan. However, the enhancement of supplements with prohibited substances is a global problem (Tsarouhas et al., 2018; Helle et al., 2019; Walpurgis et al., 2020; Duiven et al., 2021), raising concerns about the risk of unintentional ADRVs. In addition to the fact that there have been reports of health hazards due to supplement intake, it may be necessary to combine ADE methods for athletes with an education that evokes psychological aspects (Kavussanu et al., 2021) and other measures. Furthermore, we predicted that athletes with higher levels of *willingness to learn* would also have higher levels of anti-doping knowledge. However, the “strongly agree” level only had the highest percentage of correct responses to seven of the 12 ALPHA items (Q2, 5, 6, 8, 9, 11, and 12) of all the *willingness to learn* levels. When the target for the passing index was 80% or higher, most questions did not lead to a sufficient correct answer rate, except for the basic concept of anti-doping (Q1) and the responsibility to undergo doping testing (Q8). However, since *willingness to learn* is related to learning and is an essential factor in academic achievement (Schiefele, 1991), it could at least influence anti-doping knowledge. It would be worthwhile to consider adding a strategy for *willingness to learn* as a part of future initiatives in ADE. In any case, athletes lack the learning opportunities to understand anti-doping fully. Therefore, educators and organizations must provide accurate information and raise awareness beyond improving knowledge.

### Study limitations

Limitations of this study include the lack of information on exact anti-doping education taken by university athletes to date. The details of why athletes tended to be relatively more willing to learn are not precise. Future research needs to examine details of the ADE experienced by athletes in terms of the educational topics recommended by the ISE to determine which areas of learning are lacking. In addition, the ALPHA test used to measure anti-doping knowledge objectively is limited in its content. It is necessary to develop a more comprehensive scale that covers the educational topics recommended by the ISE.

## Conclusion

This study revealed the relationship between *willingness to learn* and anti-doping knowledge among Japanese university athletes. Overall, 85% of the respondents indicated they were willing to learn about anti-doping, suggesting that most Japanese university athletes are open to learning. The respondents had a high level of *willingness to learn*. However, there was no relationship between substantive *willingness to learn* and objectively measured overall anti-doping knowledge. *Willingness to learn* has been identified as one of the most important factors for academic achievement. Despite this, the lack of a significant difference in knowledge between athletes' who were willing to learn and those who were not cannot be overlooked. Furthermore, anti-doping knowledge measured by the level of *willingness to learn* was inadequate at all levels. The ISE stipulates that the acquisition of individual learning abilities and skills, based on scientific evidence, should be implemented according to the learner's stage of development. The ISE mandatory NADOs define the educational pool for ADE. NADOs and NFs will conduct mandatory ADE for RTPs and TPs, reinforcement designated, and athletes to be sent to international competitions (World Anti-Doping Agency, 2021c). WADA will audit the educational calendar and its outcomes.

On the flip side, education for non-top athletes is still considered insufficient, and the ISE recommends ADE for youth and other young athletes and in-school education. Furthermore, ISE has set the task of NADO to establish and operate an educator system to be in charge of ADE. ADE educators should be provided not only for top athletes but also for educational institutions. In Japan, Sports Agency, NADO (Japan Anti-Doping Agency), and domestic sports-related organizations have disclosed their strategic plans for establishing a single educational pathway from youth to top athletes. Given the survey results obtained in this study, educational institutions such as junior/high schools or universities should provide teaching and learning opportunities that cover anti-doping rather than leaving the task solely to NADOs and NFs. Future anti-doping research should continue to re-examine the relationship between *willingness to learn* and knowledge.

## Data availability statement

The original contributions presented in the study are included in the article, further inquiries can be directed to the corresponding author/s.

## Ethics statement

The studies involving human participants were reviewed and approved by research society Ethics Committee of the Faculty of Health and Sports Science and the Graduate School of Health and Sports Science, Juntendo University, Japan. Written informed consent for participation was not required for this study in accordance with the national legislation and the institutional requirements.

## Author contributions

YM designed this study, collected all the data, performed the statistical analysis, and prepared the manuscript. YK, SY, MN, YT, and HN supported the manuscript's preparation and gave comments according to their specialty (YK in sports psychology, SY in health psychology, MN specializes in exercise psychology, YT in orthopedics, and HN in exercise physiology). All authors read and approved the final manuscript.

## Funding

This study was supported by the Private University Research Branding Project of the Japanese Ministry of Education, Culture, Sports, Science, and Technology. It was also supported by research funding from the Institute of Health and Sports Science and Medicine, Juntendo University; the Joint Research Program of Juntendo University, Faculty of Health and Sports Science; the JSPS KAKENHI Grant Number JP20K19577 (Grant-in-Aid for Early-Career Scientists).

## Conflict of interest

The authors declare that the research was conducted in the absence of any commercial or financial relationships that could be construed as a potential conflict of interest.

## Publisher's note

All claims expressed in this article are solely those of the authors and do not necessarily represent those of their affiliated organizations, or those of the publisher, the editors and the reviewers. Any product that may be evaluated in this article, or claim that may be made by its manufacturer, is not guaranteed or endorsed by the publisher.

## Abbreviations

ADRVs: Anti-doping rule violations
WADA: World Anti-Doping Agency
ADE: Anti-doping education
ISE: International Standard for Education
NADOs: National Anti-doping Organizations
NF: National Federation
RTP: Registered Testing Pool
TP: Testing Pool.
